# Comparison of Tumor Diameter and Tumor Volume in Terms of Aggressive Tumor Behavior and Prognosis in Papillary Thyroid Cancer

**DOI:** 10.3390/cancers17081367

**Published:** 2025-04-19

**Authors:** Sevgül Fakı, Abbas Ali Tam, Nurcan İnce, Pervin Demir, Didem Özdemir, Ayşegül Aksoy Altınboğa, Oya Topaloğlu, Reyhan Ersoy, Bekir Çakır

**Affiliations:** 1 Department of Endocrinology and Metabolism, Ankara Bilkent City Hospital, 6800 Ankara, Turkey; 2 Department of Endocrinology and Metabolism, Faculty of Medicine, Ankara Yildirim Beyazit University, 6800 Ankara, Turkey; endoali2@hotmail.com (A.A.T.); dr.nurcanince@hotmail.com (N.İ.); sendidem2002@yahoo.com (D.Ö.); oyasude@gmail.com (O.T.); reyhanersoy@yahoo.com.tr (R.E.); drcakir@yahoo.com (B.Ç.); 3 Department of Biostatistics and Medical Informatics, Faculty of Medicine, Ankara Yildirim Beyazit University, 6800 Ankara, Turkey; pervin.demr@gmail.com; 4 Department of Pathology, Faculty of Medicine, Ankara Yildirim Beyazit University, 6800 Ankara, Turkey; aysegulaksoyaltinboga@aybu.edu.tr

**Keywords:** papillary thyroid cancer, lymph node metastasis, tumor volume

## Abstract

Background: Tumor diameter may not reflect tumor burden accurately in all cancers. In this study, we aimed to investigate the relationship between tumor volume (TV) and aggressive features and prognosis in papillary thyroid cancer (PTC). Methods: Patients diagnosed with single foci PTC were recruited for the study. The largest tumor diameter was considered as the primary tumor diameter. TV was calculated using the formula for an ellipsoid shape, considering the three pathologically specified dimensions. Primary tumor diameter and TV were compared in terms of aggressive tumor characteristics and prognosis. Results: The data of 118 patients were analyzed. There was no significant relationship between primary tumor diameter and lymph node metastasis (LNM), extrathyroidal extension (ETE), and vascular invasion (*p* > 0.05 for each). In patients with tumor diameter >2 cm, TV was negatively associated with LNM (*p* = 0.015). One-unit increase in TV was associated with 1.629 times greater likelihood of absence of LNM (95% CI: 1.099–2.415). When the TV was ≤5.26 cm^3^, the sensitivity and specificity for the presence of LNM were 88.9% and 75.8%, respectively. Again in this group, the sensitivity for the occurrence of ETE was 100.0% and specificity was 45.7% when the TV was ≤9.49 cm^3^. There was no significant difference in the five-year disease-free survival between tumor diameter and TV. Conclusions: In contrary to studies with other cancer types in the literature, there was a significant but negative relationship between TV and LNM. Further large-scale studies are needed to determine whether TV can be used as a prognostic factor in PTC.

## Simple Summary

Tumor diameter may not reflect tumor burden accurately in all cancers. The data of 118 patients diagnosed with single foci papillary thyroid cancer PTC were analyzed. There was no significant relationship between primary tumor diameter and lymph node metastasis (LNM), extrathyroidal extension (ETE), and vascular invasion (*p* > 0.05 for each). In patients with tumor diameter > 2 cm, tumor volume (TV) was negatively associated with LNM (*p* = 0.015). There was no significant difference in dynamic risk stratification and five-year disease-free survival between tumor diameter and TV. In contrast to studies with other cancer types in the literature, there was a significant but negative relationship between TV and LNM in our PTC cohort.

## 1. Introduction

Papillary thyroid carcinoma (PTC) is known to have an excellent prognosis and a 5-year overall survival rate of about 97%. However, there may still be some patients presenting with aggressive tumors which cause poor clinical outcomes [1]. Primary tumor diameter (TD) is one of the many risk factors that has been shown to have an impact on outcomes in PTC. Although the tumor size that is critical in terms of prognosis is unknown, a larger TD is suggested to carry a higher mortality risk from thyroid cancer. In addition, the literature provides sample data showing that a larger primary TD is associated with poor prognostic factors including multifocality, extrathyroidal growth, lymph-node metastases (LNM), or distant metastases [2].

The American Thyroid Association (ATA) guidelines strongly recommend classifying not only the risk of mortality but also the risk of recurrent and persistent disease [3]. The identification and management of recurrent and persistent disease are very important in thyroid cancer management due to the low disease-specific mortality rate. The American Joint Committee on Cancer (AJCC) staging system was primarily developed to predict the risk of mortality, and in its last edition, the age cutoff was reduced to 55 years of age, while the presence of tumor extension and distant metastasis maintained their values as prognostic factors [4]. In this system, tumors are classified as T1a, T1b, T2, and T3a according to tumor size as ≤1 cm, >1 cm and ≤2 cm, >2 cm and ≤4 cm, and >4 cm, respectively. In the revised ATA Initial Stratification System published in 2015, it was suggested that papillary microcarcinomas (PTMCs) have an excellent prognosis and that a cut-off value of 1 cm should be taken as a risk for recurrence [5].

**Figure 1:**
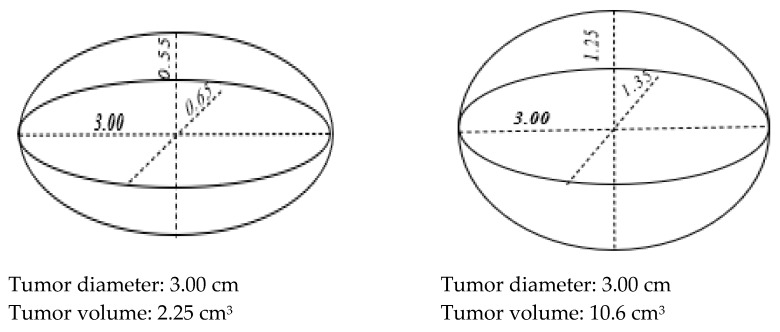
Ellipsoid calculated by tumor diameter and tumor width and height.

Even though there are factors which might influence the measurements that should be considered while comparing nodule diameter in US and tumor size in histopathology (due to the fact of: operator-dependent technique of ultrasound, the slicing the specimen can impact tumor size and change between the time of the ultrasound and surgery), thyroid ultrasound remains useful for surgical decision making and avoiding unnecessary surgeries. The TD of histopathological specimens is also the most important parameters taken into consideration during management of DTCs, it may not fully reflect the tumor burden. While the tumor volume (TV) and burden might be low in long and narrow tumors, they might be higher in those that are round in shape [6]. It can be presumed that the diameter of a spherical tumor would be more predictive for its biological behavior than the maximum diameter of an ellipsoid or a varied shape [7]. In other words, even T is the same for tumors with the same diameter, it may not actually reflect the tumor mass and tumor burden. For example, a PTC of 10.6 cm^3^ with dimensions of 3 × 1.35 × 1.25 cm and another of 2.25 cm^3^ with dimensions of 3 × 0.65 × 0.55 cm will be in the same T stage [Figure 1]. When the tumor diameter doubles, the difference in TVs will increase even more.

TV was shown to be related with poor prognosis in many types of cancer including breast cancer, laryngeal cancer, lung cancer, renal cell carcinoma, and gastric cancer [8]. However, the number of studies examining the relationship between TV and the behavior and the prognosis of the tumor in thyroid cancer are very few. To our knowledge, no study has examined the relationship between TV calculated with histopathologically determined TDs and the tumor behavior in PTC. In this study, we aimed to analyze whether three-dimensional TV is a better parameter for determining tumor behavior and prognosis compared to the one-dimensional tumor measurement in patients with PTC.

## 2. Materials and Methods

Medical records of patients who underwent thyroidectomy and diagnosed with PTC between December 2006 and January 2023 were evaluated retrospectively. To form a unique group, patients with other thyroid cancer types and patients who underwent lobectomy were excluded. Since it is not possible to determine which tumor focus was related with LNM, we also excluded patients with multifocal disease (two or more tumor foci within the thyroid gland).

In our center, all three diameters of the tumor are not routinely given in the final histopathology report. Therefore, although we had approximately 2064 patients with thyroid cancer in our database, 118 patients with all three diameters of the tumor available in reports were included in the study.

Demographic features, greatest TD, presence of lymphocytic thyroiditis, LNM, capsular invasion (CI), ETE, and vascular invasion (VI) were evaluated. CI was defined as the infiltration of the thyroid capsule by the tumor without any surrounding soft tissue or muscle involvement. ETE was defined as the invasion of surrounding soft tissue or muscle by the tumor. Central lymph node dissection (CLND) is not performed routinely in our center. The decision for central or lateral lymph node dissection is made when there are malignant or suspicious lymph nodes in preoperative examinations or intraoperative evaluation.

Dynamic risk stratification and ATA risk assessment were performed according to the 2015 ATA guidelines [3]. Local ethical committee approval was obtained in accordance with the ethical standards of the Helsinki Declaration

The eighth edition of AJCC TNM classification was used to determine the stage of PTC [4]. Greatest TD was considered as the TD. TV was calculated with the formula: length (mm) × width (mm) × height (mm) × π/6 [9,10].

### Statistical Analysis

Statistical analyses were performed using IBM SPSS Version 21.0 (Armonk, NY, USA: IBM Corp.). A *p*-value < 0.05 was accepted as statistically significant. For continuous variables, the mean ± standard deviation or median (Quartile 1–Quartile 3) was given based on the distribution of the variable. For categorical variables, frequencies (percentages) were given. The relationship between TV and TD was examined using linear and exponential regression models. The Mann–Whitney U test and Kruskal–Wallis test were used to compare two and more than two groups, respectively. For the presence of ETE, vascular, and lymph node metastasis, binary logistic regression models were constructed. For the significant model, a receiver operating characteristic (ROC) curve analysis was conducted to determine the cutoff point for the TV value. The sensitivity and specificity were calculated. Additionally, a multivariate logistic regression model was built considering age, gender, and TV as dependent variables.

## 3. Results

The mean age of the patients was 42.95 ± 12.31 years (range: 19–76), and 82.2% were female (Table 1). There were 47 (39.8%) patients with lymphocytic thyroiditis. ETE was observed in 25 (21.2%) patients, and LNM was present in 21 (17.8%) patients. Most patients (95.8%) were in Stage 1 according to the TNM staging. Dynamic risk stratification revealed an “excellent response” in 57 (67.9%) of the 84 patients with available data, and an “indeterminate response” in 17 (20.2%). According to the ATA risk classification, 6 (5.2%) of the 115 patients were classified as high risk.

### Relationship Between Tumor Diameter and Tumor Volume

**Figure 2:**
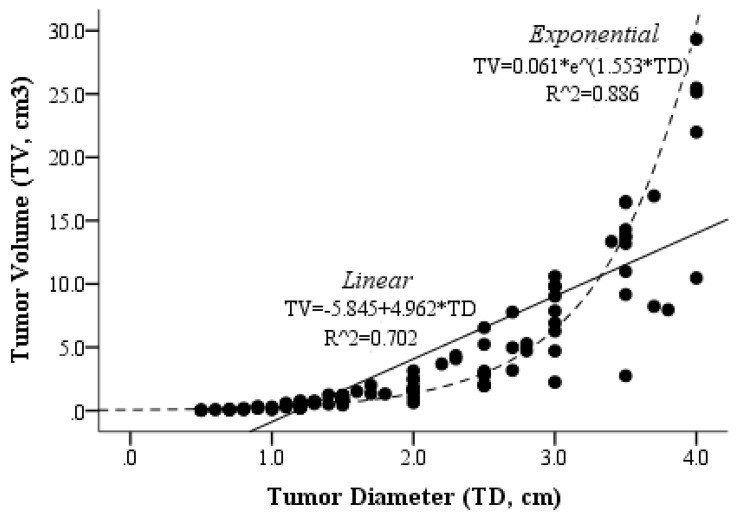
The scatter plot and curve estimation results of tumor diameter and volume.

The median TD was 1.50 cm (Q1–Q3: 1.20–2.73) and the median TV was 1.18 cm^3^ (Q1–Q3: 0.46–4.79) (Table 1). The volume distribution exhibited a positive skewness with a value of 2.275. The association and curve estimation between TD and TV are presented in Figure 2. The relationship between TD and TV was found to be better explained by an exponential model (R^2^ = 0.886). With the increase in the TD, there was a growing variance between TV and TD. The interquartile ranges (IQRs) for volume measurements were 0.88, 4.32, and 11.16 cm^3^, corresponding to tumor diameters of 2, 3, and 4 cm, respectively.

The univariate models created for determining the presence of ETE, VI and LNM by taking TV as an independent variable were not statistically significant (*p* > 0.05) (Table 2). The impact of TV was examined separately for groups classified according to TD as ≤1 cm, 1–≤2 cm, and >2 cm. Significance was identified only in the >2 cm group for the presence of LNM (*p* = 0.015). There was a negative association between TV and the presence of LNM (OR = 0.614) in this group. In other words, as the TV increased, the probability of LNM tended to decrease. The likelihood of the absence of LNM was 1.629 times greater (95% CI: 1.099–2.415) with a one-unit increase in TV.

**Figure 3:**
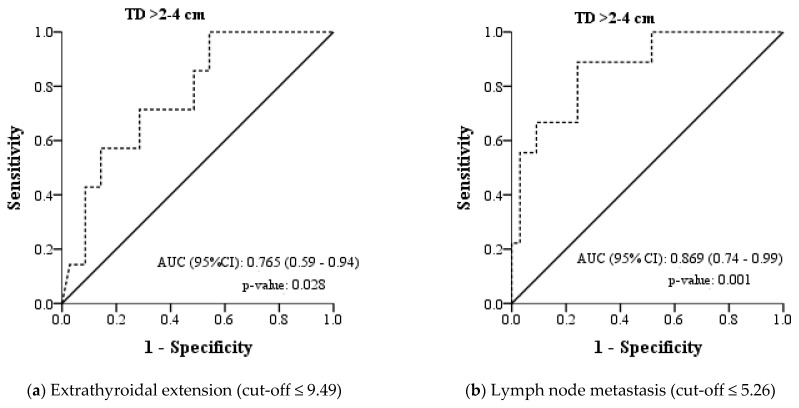
Receiver operating curve for the tumor volume as a predictor of extrathyroidal extension and lymph node metastasis.

In the univariate analysis, significance was determined in comparisons based on the presence of ETE and LNM in the TD > 2 cm group. Therefore, the ROC analysis aimed at determining the cutoff point for TV concerning these two conditions is presented in Figure 3a,b. In the TD> 2 cm group, the TV was lower in cases with the presence of ETE and LNM. Therefore, the test direction in the ROC analysis was set as a “smaller TV indicates a more positive test”. For individuals with a TD value > 2 cm, the sensitivity for the occurrence of ETE was 100.0% when the TV value was ≤9.49, with a specificity of 45.7%. A TV of ≤5.26 had a sensitivity of 88.9% and specificity of 75.8% for the presence of LNM.

The multivariate analyses using TV, age, and sex for predicting the presence of ETE, VI and LNM is shown in Table 3. For the VI and LNM, the confidence interval of OR values were wide, indicating that this result was not statistically significant (*p* > 0.05). The odds of ETE for individuals aged ≤55 was 0.290 times the odds for individuals > 55 (*p* = 0.154). The odds of ETE for males was 0.544 times the odds for females (*p* = 0.437). The odds of ETE decreased by 25.6% for each unit increase in the TV (*p* = 0.026). The interaction term suggests that the impact of TV on the odds of ETE is modified by age. For each unit increase in the TV, the odds of ETE increased by 39.1%, but this effect was modified by age (*p* = 0.042). Similarly to the TV–age interaction, the impact of TV on the odds of ETE was modified by sex. The odds of ETE increased by 31.5% for each unit increase in the TV variable, thus, this effect was modified by sex, but not statistically significant (*p* = 0.072).

The results regarding TD and TV based on 5-year disease-free survival and dynamic risk classification are presented in Table 4. The rate of individuals surviving without any disease symptoms within the 5 years after diagnosis was 76.2%. The TV was similar across risk classification categories (*p* = 0.633). Similarly, there was no difference in TV based on the 5-year disease-free survival status (*p* = 0.850).

## 4. Discussion

The stage of a cancer at the time of diagnosis is one of the most important factors that is used to predict the prognosis and determine the appropriate treatment [11]. The AJCC TNM staging system, which is widely used by clinicians, provides the standardization and accurate sharing of cancer data around the world. It evaluates local tumor extent (T), locoregional nodal spread (N), and distant metastases (M), which are the three major indicators of anatomical spread. The T category is determined by the greatest TD based on its prognostic value. The greatest TD is an easily detectable parameter that has prognostic significance for many tumors. However, it might not reflect the real tumor burden, particularly in lesions with an irregular shape [12]. Some thyroid carcinomas, as well as papillary microcarcinomas (PTMCs), may present with clinically aggressive behavior. This end led the researchers to find an alternative measurement to better evaluate histopathological specimens. In a study by Ke-cheng Jiang et al., the total tumor diameter (TTD) was found to be associated with a range of clinicopathological features, including lymph node metastasis, extrathyroidal extension, and risk stratification [13]. In another study, they mention that the risk factors for tumor recurrence in multifocal PTMCs, measured by TTD, may be more useful for the clinical management of the disease. A TTD > 10 mm was found to be a significant risk factor for lymph node metastasis (LNM). Additionally, perineural invasion, bilaterality, TTD > 10 mm, and TTD > 20 mm were also identified as significant risk factors for recurrence in this study [14].

Theoretically, the likelihood of a tumor to metastasize depends on the number of cells in the tumor and the capacity of each cell to spread. It is obvious that three-dimensional parameters such as TV can better estimate the number of cells [9]. Large tumors tend to have more tumor cells and lesser blood supply to the center. It can be suggested that, as the TV increases, there is a faster or long lasting proliferation. This means that a high TV is associated with vascular and lymphatic metastasis, and subsequently distant metastasis [15]. Since the tumor burden will be higher in a tumor with a larger volume, a more intensive treatment with higher doses of chemotherapy or radiotherapy might be required [16].

Although the T classification is divided into subgroups in recent revisions of AJCC TNM staging, the clinical course of two tumors of different sizes within the same subgroup may not be the same. For example, two PTCs of 1.1 × 0.9 × 0.7 cm and 1.9 × 1.7 × 1.5 cm are both in T1b category, but they are tumors with two different volumes and tumor burden. In addition, as the diameter of a tumor doubles (e.g., 1.5 to 3 cm or 2 to 4 cm), its volume increases by eight-fold times, resulting in a much greater increase in the number of cells than a two-fold increase.

TV was shown to be an important predictor of prognosis in several types of malignant tumors. In the study by Jiang et al. [15], tumor volume–node-metastasis (TvNM) classification was reported to be the most convenient classification for the prediction of overall survival in gastric cancer patients. In another study including patients with esophageal carcinoma, the pathological TV index was an independent prognostic factor, although TD was not predictive for survival in multivariate analysis. The authors concluded that, in patients with esophageal cancer, TD only reflects longitudinal extension, while TV is an important factor affecting the prognosis, and lymph node and systemic metastases risks are higher in tumors with high volume [17]. Su et al. [18] evaluated TD and TV in patients with stage I non-small cell lung cancer and found that both TV and greatest TD were significant prognostic factors; however, this significance was lost for TD while it remained for TV in multivariate analysis. The prognostic role of TV in thyroid cancer was investigated in very few studies. Lim et al. reported that the prognostic value of the preoperative TV for tumor recurrence in patients with T1N0 PTC was higher than that of the conventional staging system in patients older than 45 years old. In that study, 89 patients were reclassified from T1b to T1a based on their TV, and none of these patients experienced tumor recurrence. However, patients with aggressive variants, lymphovascular invasion, surrounding tissue invasion, and CI, and patients treated with postoperative radioactive iodine treatment were not included [6]. Park et al. found that TV measured by ultrasonography had a predictive value for occult central neck metastasis (OCNM). A volume of 0.385 mL (similar to that of a 9-mm sphere) was significantly and independently correlated with a higher risk of OCNM. In addition, in multivariate analysis, while tumor size was not an independent predictor for OCNM, TV was still an independent predictor [19]. In another study using ultrasonography, while TD and TV were significantly correlated with the number of LNM, they were not correlated with the presence or absence of LNM. However, TV was more closely related with the presence of LNM than TD [7].

Vikneson et al. investigated whether TV predicts lymphovascular invasion in patients with ≤2 cm DTC and observed that a larger volume was an independent predictor, whereas TD > 1 cm was not. In that study, it was found that some tumors with a 10 mm diameter had TVs greater than tumors with 11, 12, and even 20 mm diameters. However, in contrast to our study, this study included patients who had undergone hemithyroidectomy and patients with follicular and Hurthle cell thyroid cancers, which may show different spread patterns compared to PTC [10].

Active surveillance has been offered as an alternative management of low-risk PTMC in recent years [20]. During the active surveillance of papillary thyroid carcinoma, changes in tumor size were evaluated using the maximal tumor diameter. Also, the study shows that one of the most important parameters that effectively shows the progression of cancer in active surveillance studies is TV [21]. Recent studies suggest that changes in tumor volume and tumor volume doubling time (TVDT) may be considered strong dynamic markers for predicting the growth rate and progression of the tumor [22].

In our study, while there was no significant relationship between TD and aggressive tumor features, there was a significant and inverse relationship between LNM and TV in patients with TD > 2 cm. This finding does not overlap with other studies in the literature, as we discussed above. Although the pathogenesis and invasive pattern of cancer types are different, our results did not show any correlation with the studies conducted on other cancers in the literature. In this respect, questions on a controversial issue have increased. This difference in our study can actually be attributed to the limitations of our study. First of all, it is a single center and retrospective study. Although there are more than 2000 patients with thyroid cancer diagnosed and followed in our center, our sample size was smaller since three diameters are not routinely given in pathological reports. Additionally, as it is known that cystic tumors may have lower metastatic potential despite larger volumes, and our database was not suitable for distinguishing cystic nodules or cystic formation of PTC [23]. Also, it is important to note that the majority of the 118 patients in our study were in the early stages of disease (Stage 1: 113 [95.8%], Stage 2: 4 [3.4%], Stage 3: 1 [0.8%]). In this cohort, TNM staging would have likely provided more significant prognostic information, especially in higher stages. However, because this cohort was predominantly composed of patients with smaller tumors, we anticipated that comparing the tumor size and TV would yield limited insights. As TNM staging is more informative in later-stage patients with larger tumors, the inclusion of TNM staging could have compromised the generalizability of our results. Moreover, we acknowledge that the TNM system classifies patients under 55 years of age with distant metastasis as Stage 2, regardless of the tumor’s true aggressiveness. Tumor volume (TV), independent of age, provides a more precise assessment of tumor burden and aggressiveness, offering a clearer reflection of a patient’s true risk, especially in younger patients with distant metastasis. In addition, the number of patients with TD > 2 cm in which TV and LNM were related was 43 and represented a small group. Our study has low power, and we aim to validate the findings in a larger cohort. In this small group, this statistical significance was not observed in ETE and VI, we believe it may be due to interobserver variation in the pathological identification. In a study by Henry K. Su, they found that inter-pathologist agreement was the worst for invasion around thick-walled vascular structures and diagnostic for extrathyroidal extension [24].

The fact that we did not include thyroid cancer types other than PTC and patients with multifocal tumors to create a unique and homogeneous sample may have caused a selection bias. As another limitation, lymph node dissection is not routinely performed in our center, which may have affected the results. Although this ellipsoid volume formula is ideal for tumors with spherical shapes, it may be less accurate in tumors that are mostly irregular; however, the formula we prefer, as proposed in the literature, facilitates volumetric calculations based on imaging data and provides comparable results [25].

## 5. Conclusions

Tumors with the same TNM T staging might have different volumes and may show different behavioral patterns. In our study, although no significant association was found between TD and aggressive tumor characteristics, a significant inverse relationship was observed between LNM and TV in patients with a TD > 2 cm. In other words, small but mighty tumors may exhibit aggressive behavior, even in low-volume PTCs. Further studies with a larger scale, including higher stages of the disease, are required to determine whether TV should be integrated into the TNM staging system.

## Figures and Tables

**Table 1:** Demographical and clinical characteristics of patients with papillary thyroid cancer (n = 118).

Variable	Mean ± SD Median (Q1–Q3) n (%)	Variable	n (%)
**Age (years)**	**42.95 ± 12.31, 43.5 (33.0–52.0)**	**Extrathyroidal extension (ETE)**	
≤55 years	98 (83.1)	Yes	25 (21.2)
>55 years	20 (16.9)	No	93 (78.8)
**Sex**		**Vascular invasion**	
Female	97 (82.2)	Yes	21 (17.8)
Male	21 (17.8)	No	97 (82.2)
**Antithyroid antibody positivity (n= 49)**		**Lymph node metastasis**	
Positive	34 (49.3)	Yes	21 (17.8)
Negative	35 (50.7)	No	97 (82.2)
**Lymphocytic thyroiditis**		**TNM**	
Yes	47 (39.8)	Stage 1	113 (95.8)
No	71 (60.2)	Stage 2	4 (3.4)
		Stage 3	1 (0.8)
**Tumor diameter (cm)**	1.96 ± 0.99 1.50 (1.20–2.73)	**Dynamic risk stratification (DRS) (n = 84)**	
≤1 cm	19 (16.1)	Excellent response	57 (67.9)
1–≤2 cm	56 (47.5)	Biochemical incomplete response	6 (7.1)
2–≤4 cm	43 (36.4)	Structural incomplete response	4 (4.8)
**Tumor volume (cm^3^)**	3.87 ± 5.85 1.18 (0.46–4.79)	Indeterminate response	17 (20.2)
		**ATA risk (n = 115)**	
		Low risk	71 (61.7)
		Intermediate risk	38 (33.0)
		High risk	6 (5.2)

n (%): frequency (percentage), SD: Standard deviation, Q1–Q3: 25th–75th percentiles.

**Table 2:** Comparison of tumor diameter and tumor volume based on the presence of extrathyroidal extension, vascular invasion, and lymph node metastasis.

Variable	TD (cm)	TV (cm^3^)			
		All Sample (TD ≤ 4 cm)		TD > 2 cm	
	Median (Q1–Q3)	Median (Q1–Q3)	OR (95% CI)	Median (Q1–Q3)	OR (95% CI)
**Extrathyroidal extension**					
Yes	1.7 (1.23–2.5)	1.42 (0.57–2.75)	0.905 (0.796–1.028)	3.18 (2.75–7.96)	0.769 (0581–1.017)
No	1.5 (1.2–2.9)	1.05 (0.44–5.78)		8.23 (4.71–13.75)	
***p*-value**	0.823	0.901	0.125	**0.026**	0.066
**Vascular invasion**					
Yes	2.25 (1.3–3.5)	2.17 (0.64–7.9)	1.027 (0.946–1.115)	4.09 (2.74–13.19)	0.940 (0.825–1.071)
No	1.5 (1.2–2.6)	0.94 (0.44–4.72)		7.85 (4.72–13.35)	
***p*-value**	0.137	0.197	0.527	0.308	0.354
**Lymph node metastasis**					
Yes	1.85 (1.5–2.65)	1.77 (0.82–2.75)	0.921 (0.809–1.048)	2.75 (2.11–4.11)	0.614 (0.414–0.910)
No	1.5 (1.2–2.8)	0.85 (0.44–5.78)		9.03 (5.00–13.75)	
***p*-value**	0.213	0.432	0.211	**<0.001**	**0.015**

Q1–Q3: 25th–75th percentiles, TD: tumor diameter, TV: tumor volume. In the comparison of the two groups, the Mann–Whitney U test was used, and the *p*-value was provided. OR (95%CI): odds ratio (lower–upper limit of 95% confidence interval). The results of univariate logistic regression include the OR and corresponding *p*-value.

**Table 3:** Multivariate logistic regression analysis for the prediction of extrathyroidal extension, vascular invasion, and lymph node metastasis.

	Extrathyroidal Extension OR (95% CI)	Vascular Invasion OR (95% CI)	Lymph Node Metastasis OR (95% CI)
Age (≤55)	0.290 (0.053–1.590)	1.065 (0.217–5.229)	0.524 (0.100–2.739)
Sex (male)	0.544 (0.118–2.520)	0.556 (0.085–3.640)	0.285 (0.039–2.069)
TV	**0.744 (0.573–0.966)**	1.045 (0.942–1.159)	0.822 (0.660–1.024)
TV*age	**1.391 (1.011–1.913)**	0.972 (0.761–1.242)	1.249 (0.932–1.675)
TV*sex	1.315 (0.976–1.773)	0.963 (0.770–1.203)	1.220 (0.913–1.632)
Overall percentage	80.3	82.9	82.9
*p*-value of Hosmer and Lemeshow test	0.212	0.436	0.345

The *p*-value from the Hosmer and Lemeshow test was used to assess how well the model fit the observed data. A higher *p*-value suggested a good model fit. The odds ratio (OR) did not achieve statistical significance, as the confidence interval encompassed the value 1. The significant OR value was emphasized in bold. CI: Confidence interval.

**Table 4:** The comparison of the tumor diameter and tumor volume based on dynamic risk stratification and 5-year disease-free survival status (n = 84).

Variable		TD	TV
	n (%)	Median (Q1–Q3)	Median (Q1–Q3)
**Dynamic risk stratification**			
Excellent response	57 (67.9)	1.5 (1.2–3)	1.18 (0.29–5.5)
Biochemical incomplete response	6 (7.1)	1.9 (1.3–2.78)	1.03 (0.61–4.84)
Structural incomplete response	4 (4.8)	2.25 (1.63–3.25)	3.48 (1.14–8.18)
Indeterminate response	17 (20.2)	1.5 (1.1–2.6)	0.79 (0.28–3.96)
***p*-value**		0.661	0.633
**Five-year disease-free survival**			
Yes	64 (76.2)	1.55 (1.2–3)	1.21 (0.44–5.96)
No	20 (23.8)	1.85 (1.23–2.5)	1.14 (0.58–3.12)
***p*-value**		0.975	0.850

n (%): frequency (percentage), Q1–Q3: 25th–75th percentiles, TD: tumor diameter, TV: tumor volume.

## Data Availability

The original contributions presented in the study are included in the article, and further inquiries can be directed to the corresponding author.

## Abbreviations

PTC	Papillary thyroid cancer
LNM	Lymph node metastasis
ETE	Extrathyroidal extension
TV	Tumor volume
TD	Tumor diameter
ATA	The American Thyroid Association
AJCC	The American Joint Committee on Cancer
PTMC	Papillary microcarcinomas
CI	Capsular invasion
VI	Vascular invasion
CLND	Central lymph node dissection
SD	Standard deviation
OR	Odds ratio
T	Local tumor extent
N	Locoregional nodal spread
M	Distant metastases
TvNM	Tumor volume–node-metastasis
OCNM	Occult central neck metastasis
LNM	Lymph node metastasis
